# Trypanocidal Activity of Dual Redox-Active Quinones: *Trypanosoma cruzi* Mitochondrion as a Target Organelle *In Vitro* and Anti-Inflammatory Properties *In Vivo*

**DOI:** 10.3390/pathogens15010017

**Published:** 2025-12-23

**Authors:** Raquel B. Duarte, Victor F. S. Ramos, Juliana M. C. Barbosa, Gabriel M. Oliveira, Emilay B. T. Diogo, Renata G. Almeida, Alastair J. J. Lennox, Eufrânio N. da Silva Júnior, Yasmin Pedra-Rezende, Rubem F. S. Menna-Barreto

**Affiliations:** 1 Laboratório de Biologia Celular, Instituto Oswaldo Cruz, Fundação Oswaldo Cruz, Rio de Janeiro 21040-360, Brazilnumpex.victor@gmail.com (V.F.S.R.);; 2 Instituto de Ciências Exatas, Departamento de Química, Universidade Federal de Minas Gerais, Belo Horizonte 31270-901, Brazil; 3 School of Chemistry, University of Bristol Cantock’s Close, Bristol BS8 1TS, UK

**Keywords:** *Trypanosoma cruzi*, chemotherapy, naphthoquinones, mitochondrion, anti-inflammatory activity

## Abstract

Chagas disease is caused by the protozoan *Trypanosoma cruzi*, and its current treatment is limited to the use of two nitroderivatives, benznidazole (Bz) and nifurtimox; however, their toxicity often leads to discontinuation, justifying the search for new therapeutic options. The biological activity of quinones has long shown efficacy towards pathogenic microorganisms. In our previous investigations, two naphthoquinones combining ortho- and para-quinoidal moieties exhibited remarkable trypanocidal activity and presented low toxicity to host cells. Here, these two active compounds were further assessed. On trypomastigotes and epimastigotes, brominated (NQ1) and chlorinated (NQ2) nor-beta-lapachone-derived 1,2,3-triazoles were more active than Bz, presenting IC_50_/24 h values in the range of 0.8 to 3.1 µM. NQ1-treated epimastigotes showed a mitochondrial impairment and reactive oxygen species (ROS) production under electron microscopy and flow cytometry. The *in vitro* evaluation of both combinations of compounds with Bz indicated an additive interaction. *In vivo*, oral treatment with NQ1 reduced parasitemia in an acute model, with no evidence of toxicity. The treatment also led to a reduction in myocarditis, decreasing the PR interval in electrocardiographic analysis and reversing the sinus bradycardia caused by infection. These data suggest that *T. cruzi* mitochondrion are part of the NQ1 mechanism of action. *In vivo*, this compound presented moderate trypanocidal and promising anti-inflammatory activity. Its combination with Bz could enhance current therapeutic protocols and should be better explored in the future.

## 1. Introduction

Chagas disease is a neglected tropical disease, caused by the protozoan *Trypanosoma cruzi*, that is endemic in Latin America. It affects millions of people and leads to over 4000 deaths per year [1]. Due to extensive migration to well-developed countries, today, this disease is reported in all continents [2]. The life cycle of the parasite involves a hematophagous triatomine insect, a vertebrate host, and different parasitic stages: epimastigotes (insect form), trypomastigotes, and amastigotes (mammalian forms) [3]. The successful control of the triatomine vector (classical transmission) has allowed the emergence of other transmission routes. In Brazil, the great majority of new cases are related to oral transmission from the ingestion of contaminated food, including fruits—particularly açaí (*Euterpe oleracea*) and its products [4]. Regarding the course of clinical infection, two different phases can be identified in Chagas disease: an acute stage, usually asymptomatic or oligosymptomatic, characterized by the large number of trypomastigote forms easily detected in blood, and the chronic stage, which is initially asymptomatic, eventually progressing to clinical manifestations, such as cardiac, digestive, and/or neurological alterations [5,6].

Today, there are only two options for the treatment of Chagas disease: benznidazole (Bz) and nifurtimox. Both nitro derivatives are effective in acute patients, but their efficacy is drastically reduced in chronic cases, requiring long-term therapy [7]. These compounds also lead to substantial and undesirable adverse effects, which necessitates continuous multidisciplinary approaches focused on the development of alternative trypanocidal agents [8]. Studies on the therapeutic use of natural products for the treatment of parasitic diseases have been extensively reported [9,10,11,12,13,14]. Plants containing naphthoquinones have been described in traditional folk medicine for many diseases [15,16]. The biological activity of quinones, such as lapachol and beta-lapachone, was previously investigated, including their anti-inflammatory and cytotoxic properties, as well as their efficacy in tumors, viruses, and protozoa [17,18,19,20]. The classical mechanistic hypothesis for bioactive quinones is associated with their high redox potential, facilitating their action as oxidizing or dehydrogenating agents, leading to reactive oxygen species (ROS) production [21].

In the last three decades, our group has been continuously investigating the trypanocidal activity of quinones and their derivatives by screening many series, each with hundreds of compounds [19]. Some of the most active quinones and derivatives were further analyzed in relation to their mechanisms of action *in vitro* [22,23].

Four naphthoimidazoles derived from β-lapachone were extensively studied. Cell biology, biochemistry, and proteomics approaches demonstrated their activity at all *T. cruzi* stages, pointing to the parasite’s mitochondrion as the main target. It was demonstrated that the electron transport system (ETS) was severely affected by the treatment [22]. The mitochondria of trypanosomatids present unique morphological and biochemical features, making this organelle a promising target for drug intervention. In relation to redox balance, the ETS is a crucial checkpoint since it is the main source of reactive species in the parasite. Our mechanistic proposal also involved ROS production derived from ETS failure as part of the trypanocidal activity of naphthoimidazoles [22]. These compounds were also tested in acute models of Chagas disease *in vivo* but presented discrete effects and no protective effect on mortality. On the other hand, the naphthoimidazole N1 showed cardioprotective and immunomodulatory activity, justifying the analysis of its combination with Bz [24]. Regarding the mechanistic analysis of quinones, three naphthofuranquinones induced potent mitochondrial swelling in *T. cruzi* with the impairment of complex I-III activity, membrane potential collapse, a reduction in succinate-induced oxygen consumption, and hydrogen peroxide formation [19], reaffirming the parasite’s mitochondrion as a common target of quinones and derivatives [25,26,27].

In our previous studies on the chemical reactivity of naphthoquinones, we focused on the design of derivatives that combined ortho- and para-quinoidal moieties. The screening of the series on *T. cruzi* trypomastigotes showed brominated (NQ1) and chlorinated (NQ2) nor-beta-lapachone-derived 1,2,3-triazoles as the most active compounds [19]. Herein, we describe our studies regarding the assessment of the mechanisms of action of NQ1 and NQ2 on *T. cruzi in vitro*, while also extending the investigation to trypanocidal activity in an acute murine model *in vivo*.

## 2. Materials and Methods

### 2.1. Naphthoquinones

**Scheme 1:**
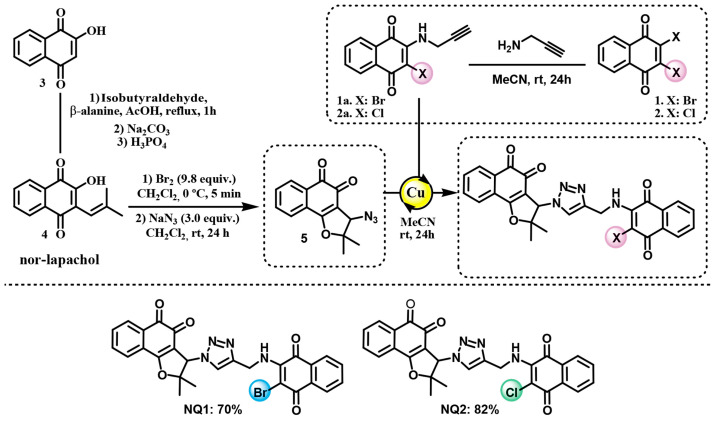
Chemical structures of the naphthoquinones NQ1 and NQ2. Note: Copper(I) iodide was used as a catalyst for the click reaction.

The synthesis of compounds NQ1 and NQ2 began with the preparation of the corresponding amino–alkyne intermediates. For NQ1, the amino–alkyne precursor was obtained from 2,3-dibromonaphthalene-1,4-dione, whereas, for NQ2, it originated from 2,3-dichloronaphthalene-1,4-dione. The nucleophilic substitution of the respective quinones with propargylamine in acetonitrile afforded the quinone–alkyne derivatives (1a and 2a). The azide coupling partner (5) required for the assembly of both target conjugates was synthesized from nor-lapachol via bromide-mediated cycloaddition, followed by nucleophilic substitution with sodium azide. This sequence furnished the desired ortho-quinone (5) in a quantitative yield. In the final step, Cu(I)-catalyzed azide–alkyne cycloaddition (CuAAC) was employed to construct the 1,2,3-triazole framework. Under standard “click” conditions, azide 5 reacted smoothly with the corresponding alkyne derivatives (1a or 2a) to deliver the triazole-bridged quinone conjugates (NQ1 and NQ2) as highly stable products (Scheme 1).

### 2.2. Parasites and Host Cells

The *T. cruzi* Y strain was used in all experiments performed. From the blood of albino outbred stock Swiss Webster mice at the peak of parasitemia, trypomastigotes were purified by differential centrifugation. Alternatively, epimastigotes were maintained in liver infusion tryptose (LIT) medium supplemented with 10% fetal bovine serum (FBS) (LGC, São Paulo, Brazil) at 28 °C, and all mechanistic assays were carried out with parasites in the exponential growth phase. Peritoneal macrophages were collected from non-infected Swiss mice (5–6 weeks) after the injection of 8 mL of RPMI medium (LGC), supplemented with 10% FBS, 1% L-glutamine (Gibco, Auckland, New Zealand), and 1% penicillin–streptomycin (10,000 units) (Gibco).

### 2.3. Trypanocidal Assays

Bloodstream trypomastigotes or epimastigotes were resuspended in a concentration of 10^7^ parasites/mL in RPMI or LIT medium, respectively. This suspension (100 μL) was added to the same volumes of naphthoquinones NQ1 and NQ2, previously prepared at twice the desired final concentrations (final dose ranges were 10.0 to 0.2 and 16.0 to 0.5 µM for trypomastigotes and epimastigotes, respectively). Incubation was performed in 96-well microplates (Nunc Inc., Rochester, NY, USA) for 24 h at 37 °C (trypomastigotes) or 28 °C (epimastigotes). Parasite quantification was performed using a Neubauer chamber, and drug efficacy was expressed by IC_50_/24 h values, which corresponded to the concentration that led to 50% lysis/proliferation inhibition of the parasite.

### 2.4. Direct Effects of the Combination of Benznidazole and Naphthoquinones on Trypomastigotes

To analyze the potential synergic activity of the two naphthoquinones NQ1 and NQ2 and Bz, the previously described fixed-ratio method was used [28]. The IC_50_/24 h values (single treatment) were previously determined in order to establish the higher concentrations, guaranteeing that the IC_50_ fell near the midpoint of a six-point two-fold dilution series using fixed-ratio solutions (5:0, 4:1, 3:2, 2:3, and 1:4 proportions). Fractional inhibitory concentrations (FICs) were employed to characterize the nature of the interaction. FIC: IC_50_/24 h of NQ1 in combination/IC_50_ of NQ1 in monotreatment. Similar calculations were applied to NQ2 and Bz. ΣFICs = FIC (NQ1 or NQ2) + FIC (Bz). ΣFICs ≤ 0.5 is considered to indicate synergism, 0.5 < ΣFICs ≤ 4.0 additive (no interaction), and ΣFICs > 4.0 antagonism. Isobolograms represent NQ1 or NQ2 FIC against Bz FIC [29].

### 2.5. Ultrastructural Analysis

Epimastigotes (5 × 10^6^ parasites/mL) were treated with 0.4–1.8 µM of the compounds NQ1 and NQ2 for 24 h in LIT medium at 28 °C. Then, the parasites were fixed with 2.5% glutaraldehyde in sodium cacodylate 0.1 M buffer (pH 7.2) at 25 °C for 40 min and post-fixed with a solution of 1% OsO_4_ containing 0.8% potassium ferricyanide and 2.5 mM CaCl_2_ in the same buffer for 30 min at 25 °C. The samples were dehydrated in an ascending acetone series and embedded in PolyBed 812 resin. Ultrathin sections were stained with uranyl acetate and lead citrate. Three independent biological replicates were examined under a JEM1011 transmission electron microscope (Jeol, Tokyo, Japan), located in the Plataforma de Microscopia Eletrônica at Instituto Oswaldo Cruz (Fiocruz).

### 2.6. Flow Cytometry Analysis

Epimastigotes (5 × 10^6^ parasites/mL) were treated with IC_50_/24 h values of NQ1 and NQ2 for 24 h, and then the mitochondrial membrane potential (ΔΨm) and ROS generation were evaluated. The probe tetramethylrhodamine (TMRE) (Sigma-Aldrich, St. Louis, MO, USA) was employed to assess ΔΨm. Parasites were incubated with 50 nM of the marker for 30 min at 28 °C, and the addition of 10 µM of the ionophore carbonyl cyanide 4-(trifluoromethoxy)phenylhydrazone (FCCP) (Sigma-Aldrich) was used for ΔΨm dissipation, minimizing unspecific labeling. TMRE data were expressed as the index of variation (IV) calculated by the equation (MT − MC)/MC, where MT is the median of fluorescence for treated parasites, and MC is that of control parasites. Negative IV values correspond to mitochondrial depolarization. For the analysis of ROS damage, epimastigotes were incubated with 10 µM dihydroethidium (DHE) (Molecular Probes, Carlsbad, CA, USA) for 30 min at 28 °C, and the addition of 20 µM antimycin (Sigma-Aldrich) was used as a positive control for production. For the DHE assay, IV was calculated by the ratio MT/MC and positive values represent the foldchange of the ROS increase. All assays were performed in a CytoFLEX S (Beckman Coulter, Franklin Lakes, NJ, EUA) in the Plataforma de Análise Multiparamétrica at Instituto Oswaldo Cruz (Fiocruz). A total of 10,000 events were acquired in the region previously established as that of the parasite.

### 2.7. In Vivo Analysis of Acute Toxicity

The no-observed-adverse-effect level (NOAEL) was calculated after the oral administration of the compound NQ1 (up to 50 mg/kg) in Swiss Webster male mice (20 to 23 g). Treated non-infected animals were monitored for toxic and subtoxic symptoms according to the Organization for Economic Co-Operation and Development (OECD) guidelines. After treatment, NOAEL values were determined, and plasma biochemical analysis was performed for alanine aminotransferase (ALT) and aspartate aminotransferase (AST), as reported previously [30].

### 2.8. Acute Infection Model and Treatment In Vivo

Male Swiss Webster mice (18–20 g) were housed with a maximum of 4 animals per cage in a specific-pathogen-free (SPF) room maintained at 20 to 22 °C under a 12/12 h light/dark cycle with 50 to 60% relative humidity and provided sterilized water and chow *ad libitum*. *T. cruzi* bloodstream trypomastigotes (Y strain) (10^4^ parasites/mice) were inoculated by the intraperitoneal route (i.p.), and parasitemia and mortality were monitored after 6 days post-infection (dpi). Parasitemia was individually quantified using the Pizzi–Brener method, analyzing 50 fields in light with an AxioLab A1 microscope (Zeiss, Oberkochen, Germany) to determine the number of parasites/mL blood [31]. Body weight was monitored throughout the whole experiment. Cumulative mortality was recorded daily, and the survival percentage was calculated. The experimental groups (8 each in non-infected group and 10 each in infected group) were as follows: non-infected and non-treated controls, non-infected and treated with 50 mg/kg NQ1, infected and non-treated controls, infected +50 mg/kg NQ1. The treatment was performed by gavage every other day, beginning at 6 dpi and ending at 10 dpi. The non-infected and infected groups received the same volume of the vehicle (6% DMSO, 3% Tween 80 in a solution 5% gum arabic in PBS—Sigma-Aldrich). At 14 dpi, muscle strength was assessed using a grip strength meter (Grip, FEP 305, Insight, São Paulo, Brazil), as previously described [32,33], and data were expressed as the mean of the strength intensity = gram-force (gf)/body weight (g). Then, all animals were euthanized and organs and plasma were collected for subsequent analysis.

### 2.9. Histopathological, Biochemical, and Cytokine Analyses

At 14 dpi, the heart, spleen, liver, and kidneys were collected and weighed. Left ventricles were collected, washed in PBS, and then included in OCT Tissue-Tek resin (Sakura, Torrance, CA, USA), frozen in liquid nitrogen, and stored at −80 °C. At least three sections (5 μm thick) of each animal were obtained in cryostat CM1850 (Leica, Wetzlar, HE, Germany), fixed in 4% paraformaldehyde (Sigma-Aldrich), and stained with hematoxylin and eosin [34]. The percentage of area occupied by cell nuclei (10 fields/sample) was quantified using the Image J software (NIH, Bethesda MD, EUA). Serum levels of AST, ALT, and the cardiac isoform of creatine kinase (CK-MB) were used as parameters for hepatic and cardiac injury. Blood samples were collected by cardiac puncture, and commercial kits were used according to the manufacturer’s instructions (LabTest Laboratory, Lagoa Santa, MG, Brazil). All absorbances were measured in a SpectraMax M3 spectrophotometer. Additionally, serum levels of different cytokines were determined: interleukin-6 (IL-6), interleukin-10 (IL-10), monocyte chemoattractant protein-1 (MCP-1), interferon-γ (IFNγ), tumor necrosis factor (TNF), and interleukin-12p70 (IL-12p70). For these analyses, a cytometric bead array mouse inflammation kit (CBA) (Becton Dickinson, Franklin Lakes, NJ, USA) was used, following the manufacturer’s guidelines (BD Biosciences, Franklin Lakes, NJ, USA). Data acquisition was performed in a CytoFLEX S flow cytometer.

### 2.10. Electrocardiographic (ECG) Analysis

At 14 dpi, the transducers were carefully placed under the skin of eachanimal, in accordance with the chosen preferential derivation (DII). Traces were recorded using a digital system (Power Lab 2/20) connected to a bioamplifier at 2 mV for 1 s (PanLab Instruments, Barcelona, Spain). The parameters analyzed were the heart rate (bpm: beats per minute), the P wave (milliseconds), and the durations of the PR, QRS, and QT intervals (milliseconds), as previously described by our group [24].

### 2.11. Ethics Statement

Swiss mice were obtained from the Institute of Science and Technology in Biomodels (ICTB) of the Oswaldo Cruz Foundation (Fiocruz). All protocols were carried out in accordance with the recommendations of the Guide for the Care and Use of Laboratory Animals of the National Council for Animal Experimentation—COBEA (http://www.cobea.org.br/) and with Federal Law 11.794 (8 October 2008) and were performed in biosafety conditions. All procedures used in this study were approved by the Animal Use Ethics Committee of IOC/Fiocruz (L-024/2023).

### 2.12. Statistical Analysis

All analyses were performed in the GraphPad Prism software, version 8.0 (GraphPad Software, La Jolla, CA, USA), and results were expressed as the mean ± SD or mean ± SEM. Data distribution was evaluated by the Shapiro–Wilk normality test. Means were compared by one-way ANOVA and the Bonferroni post test, Kruskal–Wallis test associated with Dunn’s posttest, or Mann–Whitney test as indicated in the text or the figure legends. Differences were considered statistically significant when *p* ≤ 0.05.

## 3. Results

Treatment *in vitro* with the two naphthoquinones was effective towards bloodstream trypomastigotes and epimastigotes, with IC_50_/24 h values in the range of 0.5 to 4 μM. For trypomastigotes, the IC_50_/24 h values were 0.8 ± 0.2 and 3.1 ± 0.7 μM for NQ1 and NQ2, respectively, in the absence of blood. The proliferative forms showed the same susceptibility to NQ1 as the infective forms, but NQ2 was more active in epimastigotes, with IC50/24 h values of of 1.8 ± 0.1 μM (Table 1). In relation to the clinical drug Bz, both naphthoquinones exhibited higher activity in all experimental conditions tested. Without blood, the IC_50_/24 h values for trypomastigotes were 12.2- and 3.3-fold lower for NQ1 and NQ2, respectively. In proliferative form, a similar result was observed, and the IC_50_/24 h was 15.2- and 12.6-fold lower than the Bz value (Table 1).

**Figure 1:**
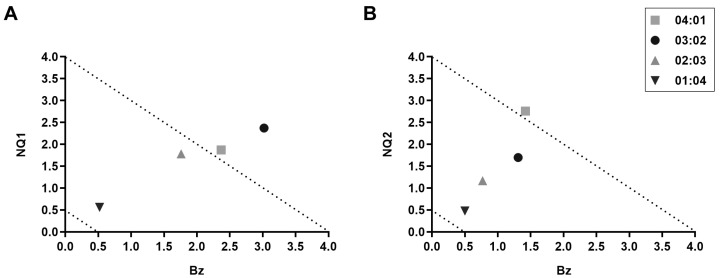
*In vitro* interactions between naphthoquinones and Bz in *T. cruzi* bloodstream trypomastigotes. Isobolograms of (**A**) NQ1 and (**B**) NQ2.

Employing the previously described fixed-ratio method [28], the interaction between Bz and the two naphthoquinones was classified on bloodstream trypomastigotes. The interaction of Bz and NQ1 was additive (no interaction) (∑FIC = 3.56) (Table 2). In the proportions 2:3 and 1:4, the interaction was considered additive, while, in the proportions 4:1 and 3:2, they were classified as antagonistic (Figure 1A). In relation to NQ2, the interaction with Bz was also classified as additive (∑FIC = 2.53). Among the tested proportions, the 1:4 ratio exhibited the lowest ∑FIC (0.98), indicating that it came the closest to a synergistic effect (Table 3). In the proportions 3:2, 2:3, and 1:4, the interaction was additive, and the proportion 4:1 indicated antagonism (Figure 1B).

**Figure 2:**
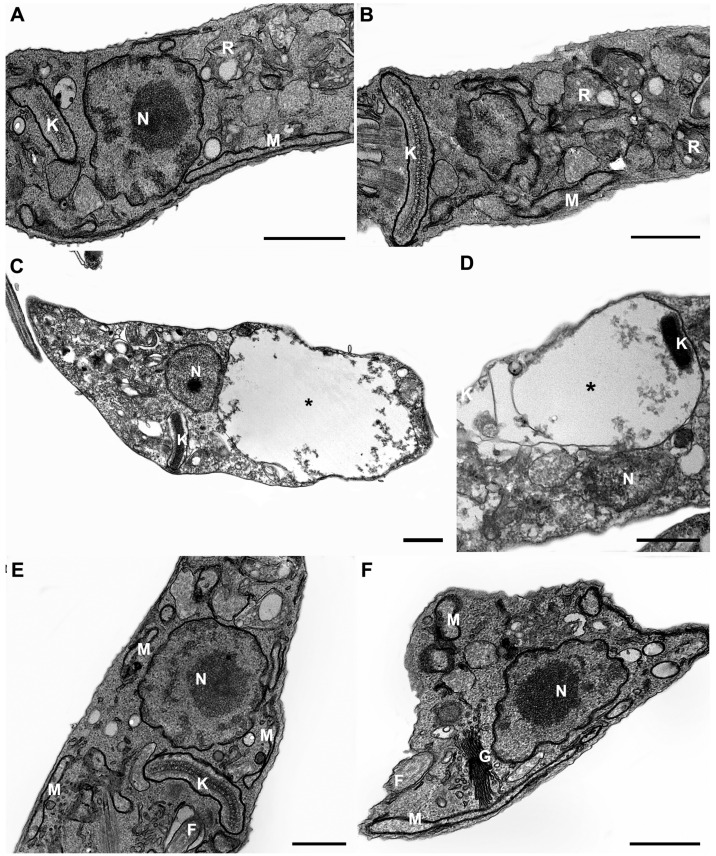
Ultrastructural analysis of *T. cruzi* epimastigotes treated with 0.8 µM of NQ1 and 1.8 µM of NQ2 *in vitro*. (**A**,**B**) Untreated epimastigotes showing typical morphology of nucleus (N), mitochondrion (M), reservosomes (R), and kinetoplast (K). (**C**,**D**) Treatment with NQ1 induced remarkable mitochondrial swelling (black asterisks). (**E**,**F**) NQ2-treated parasites presented no ultrastructural damage. F: flagellum; G: Golgi. Bars = 0.5 µm.

The evaluation of the ultrastructural damage of NQ1 and NQ2 was investigated in epimastigotes treated for 24 h, which were analyzed at the IC_50_ dose and half of this concentration. Transmission electron microscopy analysis revealed a control parasite presenting normal morphological aspects of organelles, such as the mitochondrion, nucleus, reservosomes, and kinetoplast (Figure 2A,B). At the IC_50_ concentration, NQ1 induced strong mitochondrial swelling, with loss of cristae and matrix electron density, showing a washedout phenotype (Figure 2C,D). On the other hand, the treatment of epimastigotes with NQ2 at the IC_50_/24 h presented no ultrastructural injury (Figure 2E,F), showing all morphological aspects similar to untreated parasites. The twonaphthoquinones did not promote any relevant damage detected by electron microscopy at half of the IC_50_ dose (Figure S1).

**Figure 3:**
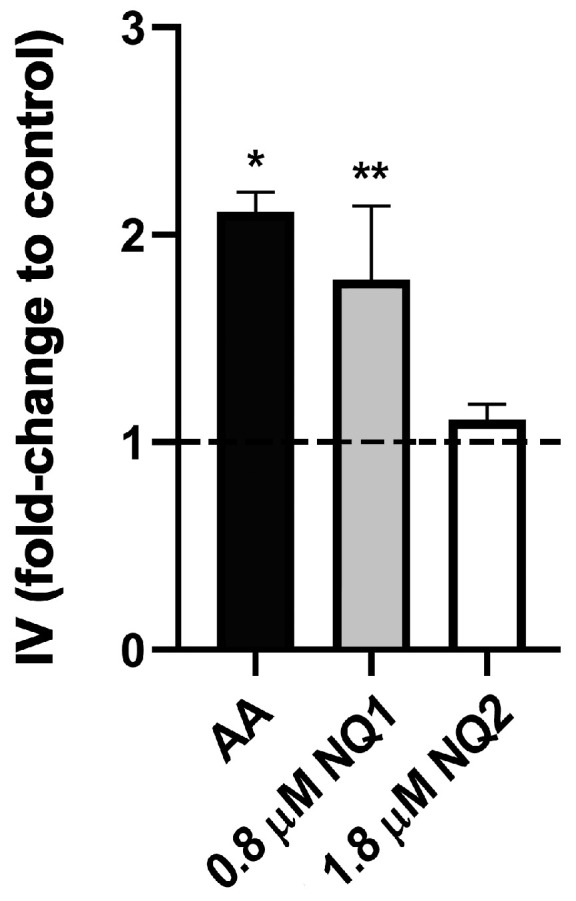
ROS analysis in *T. cruzi* epimastigotes treated with naphthoquinones *in vitro*. The data were expressed as the index of variation (foldchange to control), calculated using the ratio of DHE median fluorescence for treated and control parasites. Dashed line represents untreated controls. AA: antimycin A. The data are represented as the mean ± SEM of at least three independent experiments. Asterisks indicate significant differences in relation to untreated control group (* *p* ≤ 0.0001; ** *p* ≤ 0.0033) according to one-way ANOVA test.

The ΔΨm was analyzed by the monitoring of TMRE labeling in flow cytometry. The data were normalized by using the medians of all experimental conditions after FCCP addition. As was observed in the ultrastructural analysis, the lower concentrations of both compounds tested (half of the IC_50_/24 h), and the higher dose of NQ2, led to no reduction in the fluorescence intensity of the marker. Only NQ1 at 0.8 µM (IC_50_) induced a significant 80% decrease in TMRE labeling (Table 4). Additionally, ROS production was assessed by DHE labeling, using the mitochondrial complex III inhibitor AA as a positive control for generation. NQ1 led to a remarkable increase in probe fluorescence, being 78% (0.8 µM) higher than in untreated parasites. NQ2 did not promote an increase in DHE labeling at the IC_50_ dose (Figure 3).

**Figure 4:**
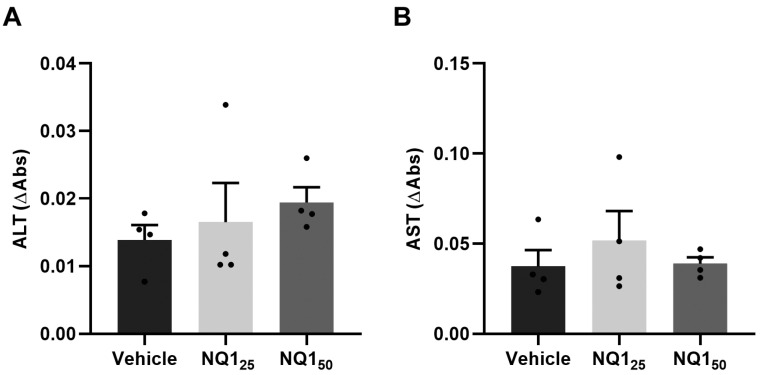
Biochemical analysis of acute toxicity of the naphthoquinone NQ1 *in vivo*. Non-infected animals were treated orally with 5 doses of NQ1 up to 50 mg/kg. Plasma levels of (**A**) ALT and (**B**) AST. Mean ± standard deviation of four animals per group. ALT: alanine aminotransferase; AST: aspartate aminotransferase; NQ1_25_: cumulative dose of 25 mg/kg; NQ1_50_: cumulative dose of 50 mg/kg.

**Figure 5:**
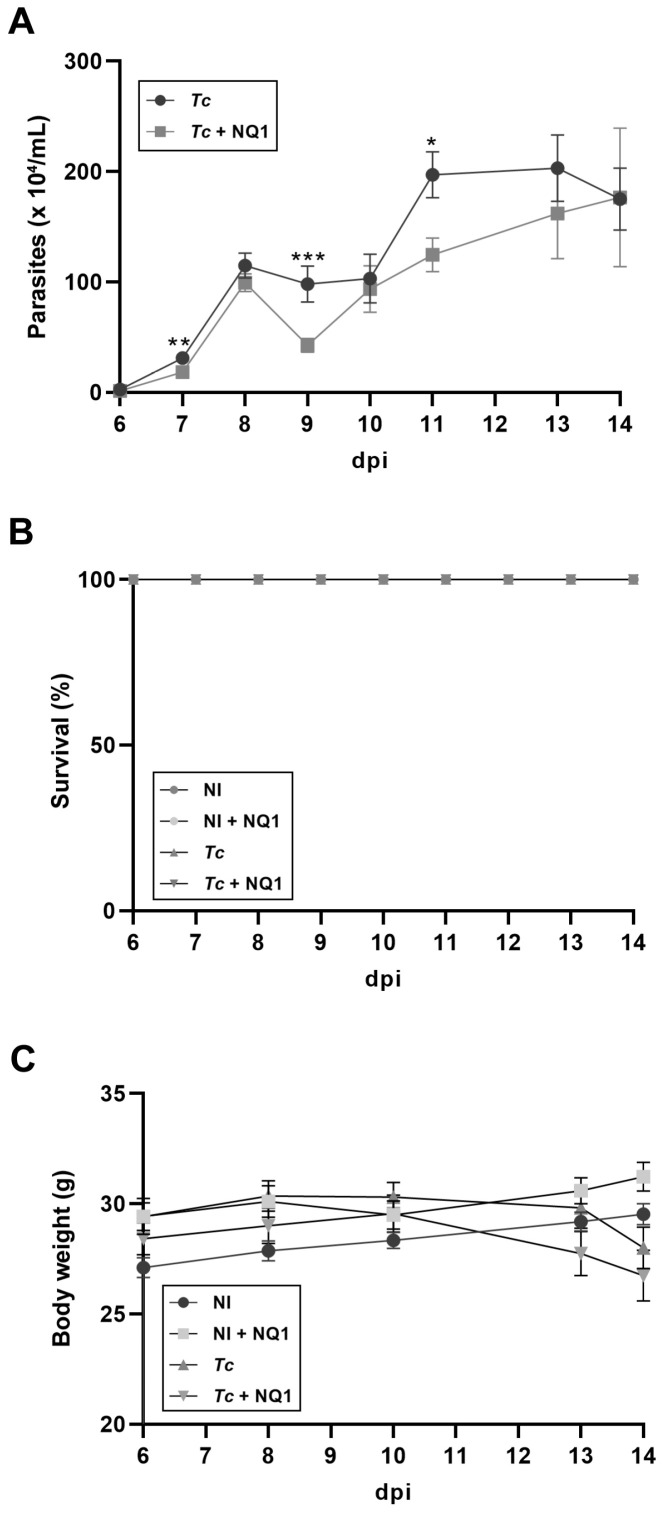
Effects of NQ1 (50 mg/kg) on the course of *T. cruzi* acute infection. (**A**) Parasitemia. Significant differences between the infected and treated groups were detected at 7, 9, and 11 dpi. (**B**) Survival. (**C**) Body weight. No mortality and weight alterations were detected in untreated or treated groups. Mean ± standard deviation of at least 8 animals per group. Asterisks indicate significant differences between untreated and treated groups (* *p* < 0.05; ** *p* < 0.01; *** *p* < 0.001) according to Mann–Whitney test. NI: non-infected; Tc: *T. cruzi*; dpi: days post-infection.

In the next step, NQ1 was synthesized in large amounts to evaluate its trypanocidal activity *in vivo*. First, the acute toxicity of the compound in non-infected animals was investigated. Treatment with 25 and 50 mg/kg induced no side effects. The analysis of the biochemical markers of hepatic damage (AST and ALT) presented no significant differences in relation to the control group that received only the vehicle (Figure 4). As the highest concentration did not lead to undesirable symptoms, 50 mg/kg was the dose selected for the subsequent assays. Using the acute infection model (Y strain, inoculum 10^4^ trypomastigotes), 50 mg/kg NQ1 was administered by gavage (five doses from 6 to 10 dpi). The naphthoquinone significantly reduced bloodstream parasitemia at 7, 9, and 11 dpi in comparison with the untreated group (Figure 5A). Up to 14 dpi, no mortality or significant variation in body weight was detected in all experimental groups (Figure 5B,C).

At 14 dpi, all animals were euthanized and other analyses were performed. Firstly, the weights of some crucial organs were evaluated (Figure S2). The infection induced a hepatosplenomegaly profile, with a spleen that was almost four-fold larger than in non-infected animals and a liver with a relative weight that was at least 15% higher (Figure S2B,C). On the other hand, the heart and kidneys presented similar relative sizes among all groups analyzed (Figure S2A,D). No differences were observed between the untreated and treated infected groups (Figure S2A–D). The analysis of muscular strength also did not demonstrate any variation among the four groups studied (Figure S3).

**Figure 6:**
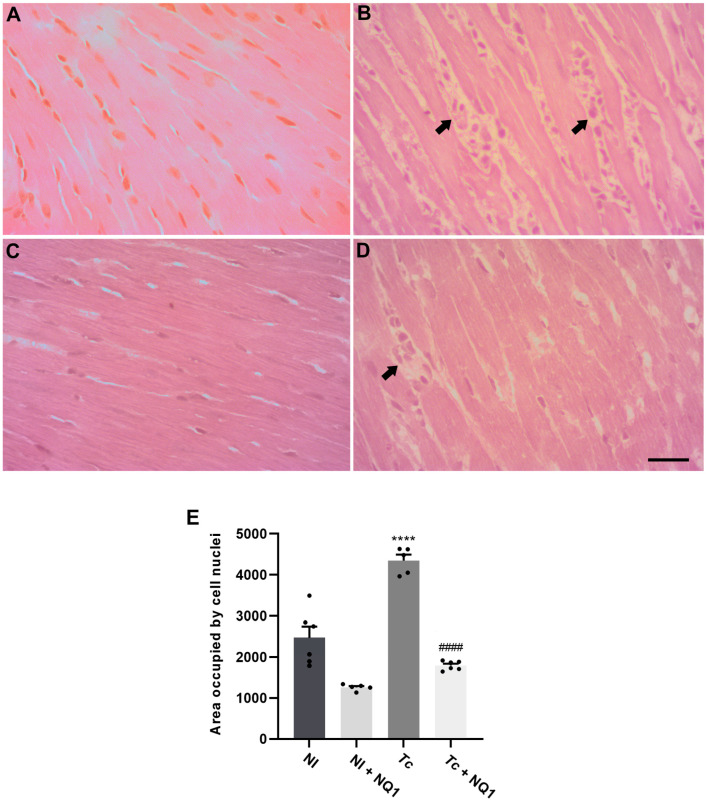
Effects of NQ1 (50 mg/kg) on cardiac inflammation at 14 dpi. (**A**–**D**) Representative histological images (hematoxylin and eosin staining). (**A**) NI; (**B**) Tc; (**C**) NI NQ1; (**D**) Tc + NQ1. Black arrows indicate inflammatory infiltrates. Bar = 100 µm. (**E**) Quantification of inflammatory infiltrates. Mean ± standard deviation of at least 8 animals per group. Asterisks indicate significant differences between non-infected and infected groups (**** *p* < 0.0001); hashtags indicate significant differences between untreated and treated groups (^####^ *p* < 0.0001). The statistical test employed was one-way ANOVA. NI: non-infected; Tc: *T. cruzi*; dpi: days post-infection.

**Figure 7:**
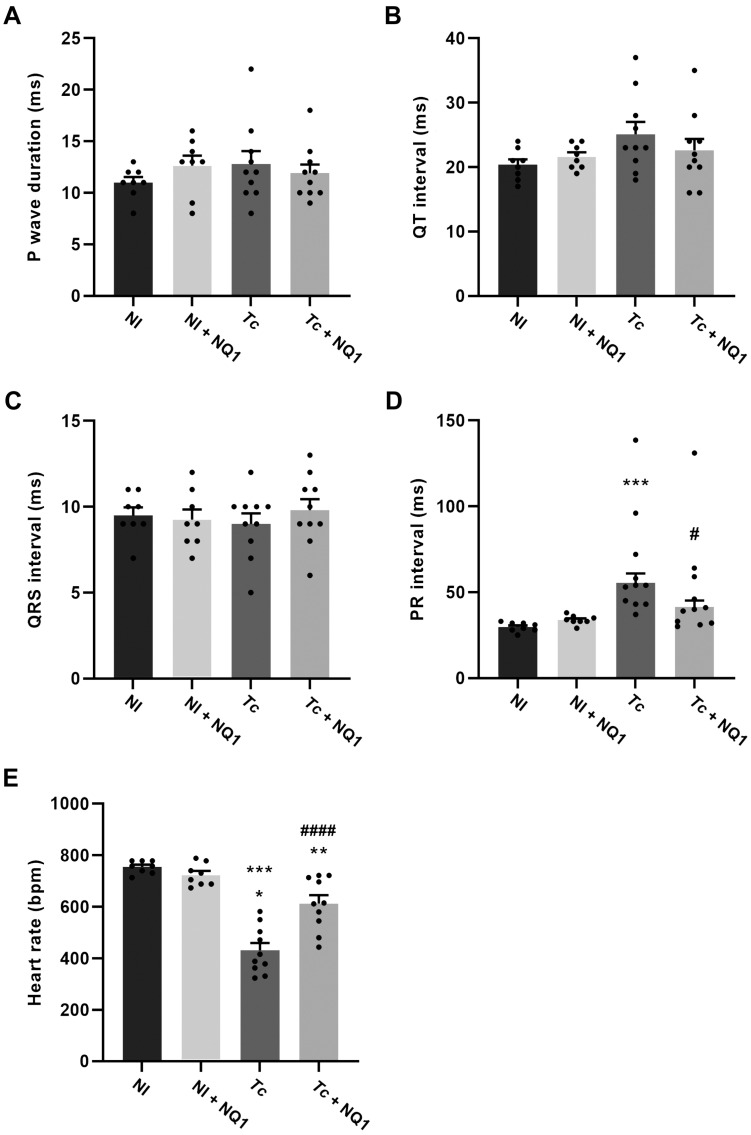
Effect of NQ1 (50 mg/kg) on electrocardiographic profile at 14 dpi. (**A**) P wave. (**B**) QT interval. (**C**) QRS interval. (**D**) PR interval. (**E**) Heart rate. Mean ± standard deviation of at least 8 animals per group. Asterisks indicate significant differences between non-infected and infected groups (* *p* < 0.05; ** *p* < 0.01; *** *p* < 0.001). Hashtags indicate significant differences between untreated and treated groups (^#^ *p* < 0.05; ^####^ *p* < 0.0001). All data were analyzed by one-way ANOVA test. NI: non-infected; Tc: *T. cruzi*; dpi: days post-infection.

To investigate the possible effects of the treatment on myocarditis, a histo pathological analysis was performed at 14 dpi (Figure 6). The tissues of non-infected animals (treated or not) showed typical morphological aspects of the fibers, and no inflammation was observed (Figure 6A,C,E). Extensive inflammatory infiltrates were detected in infected mice (Figure 6B), but this number was reduced by NQ1 treatment (Figure 6D). The quantification of the percentage of area occupied cellular nuclei confirmed the qualitative analysis, demonstrating an 80% increase in the inflammation area in the infected group, a phenotype protected by the treatment (Figure 6E). To assess heart functionality, an ECG analysis was also performed at 14 dpi (Figure 7). Infection induced an 86% increase in the PR interval (Figure 7D) but no other electric alterations (Figure 7A–C). Such increases in the PR interval were reversed in the infected group treated with NQ1 (Figure 7D). Additionally, infection led to bradycardia, but treated animals showed partial recovery in this parameter (Figure 7E).

**Figure 8:**
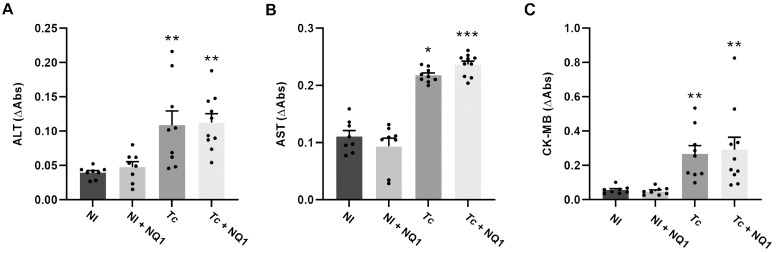
Effects of NQ1 (50 mg/kg) on the plasma levels of biochemical markers at 14 dpi. (**A**) ALT, (**B**) AST, (**C**) CK-MB. Mean ± standard deviation of at least 8 animals per group. Asterisks indicate significant differences between non-infected and infected groups (* *p* < 0.05; ** *p* < 0.01; *** *p* < 0.001) according to one-way ANOVA and Kruskal–Wallis test. ALT: alanine aminotransferase; AST: aspartate aminotransferase; CK-MB: cardiac isoform of creatine kinase; NI: non-infected; Tc: *T. cruzi*; dpi: days post-infection.

Once the inflammation was reduced in treated animals, plasma inflammatory cytokine profiles were assessed at 14 dpi. As was expected, increased levels of MCP-1, IL-10, IFN-ɣ, TNF, and IL-12p70 were observed in the infected group, but the treatment induced no differences in the levels of the cytokines analyzed (Figure S4). The biochemical analyses evidenced a remarkable increase in the levels of ALT, AST, and CK-MB in the infected groups, suggestive of hepatic and cardiac lesions. NQ1 could not restore these parameters after infection (Figure 8A–C).

## 4. Discussion

The limited efficacy and undesirable adverse effects of the currently available treatments highlight the need for continuous multidisciplinary efforts to develop alternative drugs for Chagas disease [8]. Natural naphthoquinones are widely spread among several botanical families, and they are considered beneficial in medicinal chemistry because of their biological activity and structural properties [37,38]. Our group has been investigating the trypanocidal activity of naphthoquinones and derivatives for the last 30 years, synthesized based on molecular hybridization approaches. Nor-β-lapachone-based 1,2,3-triazoles have been designed to enhance the biological activity of the naphthoquinone via the addition of the pharmacophoric group 1,2,3-triazole [19]. It is well known that the triazole nucleus or a cyclic dienone moiety presents a large variety of biological activity, such as microbicidal, antiviral, anticancer, and anti-inflammatory activity [39,40,41].

Previous reports on *T. cruzi* trypomastigotes indicated that NQ1 and NQ2 were the most active naphthoquinones among a series of nor-β-lapachone derivatives in the presence of 5% blood at 4 °C (IC_50_/24 h values of 6.8 ± 0.7 and 8.2 ± 0.7 µM, respectively), as they were at least 12-fold more effective than Bz under the same experimental conditions. In this previous study, the toxicity to the host cells was also assessed. The LC_50_/24h values were 63.1 and 281.6 µM for NQ1 and NQ2, producing selectivity index (SI) values of 9.3 and 34.3 [19]. Here, the trypanocidal activity of both compounds was further investigated. In the absence of blood at 37 °C, the effect on bloodstream forms increased, demonstrated by IC_50_/24 h values reduced to 0.8 ± 0.2 (NQ1) and 3.1 ± 0.7 µM (NQ2), representing an 8.5- and 2.6-fold increase, respectively. The presence of blood clearly reduced the trypanocidal efficacy, as previously reported for β-lapachone and other naphthoquinones, reinforcing the hypothesis that the bioavailability of these compounds is compromised by their interaction with serum proteins [42]. Recalculating the SI values (ratio LC_50_/IC_50_) using the IC_50_ values obtained at the temperature of the mammalian host (37 °C), the values strongly increased to 78.9 and 90.8, respectively. These two compounds also showed better activity than Bz, with 12.2- (NQ1) and 3.3-fold (NQ2) higher activity in the absence of blood. For mechanistic purposes, the effect on epimastigotes was also tested. The IC_50_/24 h values were 0.8 ± 0.2 (NQ1) and 1.8 ± 0.1 µM (NQ2), and this activity was also at least 12-fold higher than that of Bz in these parasite forms. In a comparison with another active nor-β-lapachone-based 1,2,3-triazole previously studied by our group, NQ1 and NQ2 showed greater effects on epimastigotes and trypomastigotes [23]. These promising data led to the evaluation of the possible synergetic activity of NQ1 or NQ2 and Bz *in vitro* via the fixed-ratio method [28]. Unfortunately, our results pointed to no synergic effect. For both naphthoquinones, the recurrent profile showed no interaction, failing to justify any further investigation in this direction.

For the assessment of the mechanism of action, the epimastigote form was chosen as a model due to its proliferative and axenic profile and to avoid any experimental interference. For all mechanistic analyses, the doses of the two naphthoquinones used never exceeded the IC_50_/24 h so as to allow the identification of primary targets. Firstly, an electron microscopy technique was employed to identify ultrastructural damage caused by the compounds in the parasite. Surprisingly, remarkable mitochondrial swelling was detected only in NQ1-treated parasites at the IC_50_ concentration. No morphological alterations were detected after treatment with NQ2. Corroborating these data, our flow cytometry analysis indicated that only epimastigotes treated with the IC_50_ of NQ1 presented a significant loss in ΔΨm (a decrease of 80% in TMRE labeling). A ΔΨm decrease has been extensively reported in *T. cruzi* treated with different quinones and derivatives [22,27]. The mitochondrion in trypanosomatids is a unique organelle as it presents morphological and metabolic peculiarities. Its distinct characteristics make this organelle an attractive target for chemotherapeutic strategies [22].

Indeed, the mitochondrial role as a drug target is welldocumented for compounds with redox properties, since the organelle is the main source of ROS in the parasite. Quinones commonly show high redox potential, being easily reduced, and this electrochemical behavior has been related to their biological activity. Previous studies have reported a similar phenotype in *T. cruzi* epimastigotes treated with different naphthoquinones and derivatives; this was also associated with a strong reduction in respiratory rates and the increased formation of mitochondrial ROS [22,43,44]. Treatment with NQ1 led to a significant increase in ROS production, demonstrated by DHE labeling analyzed in flow cytometry. Such a remarkable increase had already been observed in the IC_50_ concentration, and it was quite similar to that detected in AA-treated parasites. AA is a potent inhibitor of ETS complex III, extensively described as the main source of ROS in trypanosomatids. The similar ROS levels detected only reinforced the intensity of the phenotype triggered by NQ1.

The opposite phenotypes were observed in NQ1- and NQ2-treated parasites in the mechanistic analysis, reinforcing the notion that small differences in chemical structure lead to distinct biological activity. In this case, the substitution of bromine (NQ1) with chlorine (NQ2) at the same position culminated in the absence of mitochondrial dysfunction. In *para*-quinones, this halogen substituent can govern nucleofugality. Owing to the higher polarizability and weaker C-Br bond, bromine generally exhibits superior leaving-group abilities compared to chlorine. Accordingly, *para*-bromoquinones are intrinsically more susceptible to nucleophilic attacks by physiological nucleophiles than their chloro-analogs. Indeed, our group previously analyzed the mechanism of action of another 1,2,3-triazole derived from nor-β-lapachone, and our evidence pointed to autophagy, especially of reservosomes, as well as mitosis blockage and ROS generation, independently of the mitochondrion, which suggests an alternative mechanism for trypanocidal activity. It was suggested that the presence of the triazolic moiety led to an alteration in structural configuration and ROS production but decreased the drug availability in the mitochondrion [23]. Nonetheless, the total absence of an ultrastructural and flow-cytometric phenotype in NQ2-treated parasites will help us to further evaluate its mechanisms through other approaches.

The strong trypanocidal activity together with the low toxicity of both naphthoquinones *in vitro* encouraged us to perform analyses in an animal model. For this set of experiments, large quantities of NQ1 were synthesized, allowing a preliminary investigation of its activity *in vivo* in an acute model of Chagas disease. NOAEL and the biochemical evaluation (ALT and AST) of treated non-infected animals demonstrated that the highest dose tested (50 mg/kg) showed no acute toxicity, and this concentration was selected for the subsequent analysis under infection. The oral administration of five doses of 50 mg/kg NQ1 (after positive parasitemia) induced a moderate reduction in parasitemia levels at 7, 9, and 11 dpi, but no significant impact was observed on the mortality rate. Our most prominent result was related to cardiac dysfunction. Treatment with the naphthoquinone led to a strong decrease in the inflammatory infiltrate area in the heart tissue to uninfected control levels. NQ1 also significantly reduced the PR interval in the ECG analysis, and it partially reversed sinus bradycardia. Such reductions in prolonged PR intervals and increased heart rates are suggestive of a reversed atrioventricular block and arrhythmia, respectively [45]. Despite the improvement in myocarditis and ECG parameters, the cardiac damage evidenced by increased CK-MB levels was still unaltered. Echocardiography analysis should be performed to further characterize the protective effects of the compound in heart physiology.

Previously, our group investigated the activity of three naphthoimidazoles derived from beta-lapachone and observed very similar data. Using the same acute model (Swiss Webster mice, Y strain, inoculum 10^4^ trypomastigotes) and therapeutic protocol (five doses, 100 mg/kg, orally), naphthoimidazole N1 also moderately reduced parasitemia and heart inflammation, partially reversing sinus bradycardia, but mortality rates were not affected [24]. Despite the huge differences between N1 and NQ1’s chemical structures, the anti-inflammatory properties, as well as the discrete trypanocidal activity, were quite the same. It is important to mention that the dose of N1 administered was two-fold higher than that of NQ1 and led to important neurological adverse effects. The absence of clear side effects and the lower concentrations used strongly motivate the further analysis of NQ1 in alternative therapeutic approaches, including combinations with Bz or even new formulations of NQ1. The use of atenolol and propranolol (beta-blockers) in combination with amiodarone or other anti-arrhythmic drugs has been extensively reported to treat heart dysfunction [46].

A few other studies analyzing the effects of quinones and derivatives in murine *T. cruzi* infection have been performed. The treatment of trypomastigotes with allyl-beta-lapachone *in vitro* was effective in impairing parasite infectivity *in vivo* [42]. Using an abortive protocol for treatment, before the seropositivity of the animals, 3-diphenyl-1,4-naphthoquinone and aryloxy-quinones also presented efficacy *in vivo* [47,48]. In these three studies, the protocol employed was significantly different from ours, which makes it challenging to compare their efficacy. On the other hand, a similar therapeutic approach was used for abietane 1,4-benzoquinone (20 mg/kg, five doses orally). The results were very promising, showing an effect on parasitemia that was similar to that of Bz, with no significant alterations in biochemical markers; however, PCR still revealed parasite stocks in almost all tissues analyzed [27]. Further cardiac analyses of animals treated with this quinone must be performed, as heart failure is the main cause of death in Chagas disease. In this direction, combined treatment with NQ1 and abietane 1,4-benzoquinone could be investigated.

## 5. Conclusions

In summary, both quinones presented potent trypanocidal activity (low micromolar range) under all experimental conditions, with low toxicity and higher efficacy than the clinical drug *in vitro*. The mechanistic analysis pointed to the mitochondrial impairment and ROS production as part of the trypanocidal action of NQ1. This naphthoquinone also showed moderate trypanocidal and promising anti-inflammatory activity *in vivo*. New tests with alternative formulations, as well as the improvement of therapeutic protocols, could be attractive options for the treatment of affected individuals. Furthermore, additional pharmacological and other experimental evaluations must be performed before clinical trials are conducted.

## Figures and Tables

**Table 1:** IC_50_/24 h values (µM) of naphthoquinones NQ1 and NQ2 on *T. cruzi*.

Compound	Bloodstream Trypomastigote		Epimastigote
	5% Blood, 4 °C	0% Blood, 37 °C	
NQ1	6.8 ^1^ ± 0.7	0.8 ^3^ ± 0.2	0.8 ^3^ ± 0.2
NQ2	8.2 ^1^ ± 0.7	3.1 ^3^ ± 0.7	1.8 ^3^ ± 0.1
Bz	103.6 ^2^ ± 0.6	9.7 ^4^ ± 2.3	17.1 ^3^ ± 0.5

^1^ [19]. ^2^ [35]. ^3^ Mean ± standard deviation of at least three independent experiments. ^4^ [36].

**Table 2:** Trypanocidal effects of the combination of Bz and NQ1 on *T. cruzi* bloodstream trypomastigotes.

Bz–NQ1	FIC Bz	FIC NQ1	∑FIC
4:1	1.87 ^1,2^ ± 0.57	2.37 ± 0.86	4.24
3:2	2.37 ± 0.93	3.02 ± 0.45	5.39
2:3	1.78 ± 0.48	1.76 ± 0.56	3.54
1:4	0.56 ± 0.32	0.52 ± 0.01	1.09
Mean of ∑ in combination	1.65	1.92	3.56

^1^ Data expressed as fractional inhibitory concentrations (FICs) and ∑FIC means. ^2^ Mean ± standard deviation of three independent experiments.

**Table 3:** Trypanocidal effects of the combination of Bz and NQ2 on *T. cruzi* bloodstream trypomastigotes.

Bz–NQ2	FIC Bz	FIC NQ2	∑FIC
4:1	2.76 ^1,2^ ± 2.04	1.42 ± 0.53	4.18
3:2	1.7 ± 0.73	1.31 ± 0.45	3.00
2:3	1.17 ± 0.11	0.77 ± 0.17	1.94
1:4	0.48 ± 0.12	0.5 ± 0.43	0.98
Mean of ∑ in combination	1.53	1.00	2.53

^1^ Data expressed as fractional inhibitory concentrations (FICs) and ∑FIC means. ^2^ Mean ± standard deviation of three independent experiments.

**Table 4:** Flow cytometry analysis of ΔΨm in *T. cruzi* epimastigotes.

	Dose (µM)	Median	IV ^1^
Control	-	34,565.3 ± 4172.8 ^2,3^	0.0
NQ1	0.4	34,520.1 ± 2808.7	0.01
	0.8	6778.1 ± 2082.5 *	−0.80
NQ2	0.9	35,367.2 ± 4865.6	0.02
	1.8	37,430.2 ± 3744.2	−0.05

^1^ IV = (MT − MC)/MC, where MT corresponds to the median fluorescence for treated parasites, and MC corresponds to that of control parasites. ^2^ Each experimental condition was normalized by incubation with FCCP. ^3^ Mean ± standard deviation of three independent experiments. * Asterisk indicates significant difference in relation to the untreated control group (*p* ≤ 0.0001) according to one-way ANOVA test.

## Data Availability

The data that support the findings of this study are not publicly available due to privacy and ethical restrictions. However, they can be obtained from the corresponding author upon reasonable request. Data sharing is subject to the approval of the institutional ethics committee and may require a data use agreement.

## Supplementary Materials

The following supporting information can be downloaded at https://www.mdpi.com/article/10.3390/pathogens15010017/s1, Figure S1: Ultrastructural analysis of *T. cruzi* epimastigotes treated with 0.4 µM NQ1 and 0.9 µM NQ2 *in vitro*; Figure S2: Effects of NQ1 (50 mg/kg) on organ weights at 14 dpi; Figure S3: Effects of NQ1 (50 mg/kg) on muscular strength at 14 dpi; Figure S4: Effects of NQ1 (50 mg/kg) on plasma levels of cytokines at 14 dpi.

## Abbreviations

The following abbreviations are used in this manuscript:

Bz	Benznidazole
NQ1	Brominated nor-beta-lapachone-derived 1,2,3-triazole
NQ2	Chlorinated nor-beta-lapachone-derived 1,2,3-triazole
ROS	Reactive oxygen species
ETS	Electron transport system
FBS	Fetal bovine serum
IC_50_	Concentration that led to 50% lysis/proliferation inhibition of parasite
LC_50_	Concentration that led to 50% lysis/proliferation inhibition of host cells
SI	Selectivity index
FIC	Fractional inhibitory concentration
TMRE	Tetramethylrhodamine
ΔΨm	Mitochondrial membrane potential
FCCP	Carbonyl cyanide 4-(trifluoromethoxy)phenylhydrazone
DHE	Dihydroethidium
AA	Antimycin A
NOAEL	No-observed-adverse-effect level
ALT	Alanine aminotransferase
AST	Aspartate aminotransferase
dpi	Days post-infection
CK-MB	Cardiac isoform of creatine kinase
IL-6	Interleukin-6
IL-10	Interleukin-10
MCP-1	Monocyte chemoattractant protein-1
IFNγ	Interferon-γ
IL-12p70	Interleukin-12p70
TNF	Tumor necrosis factor
CBA	Cytometric bead array
ECG	Electrocardiograph
