# Supplementary material for: Trypanocidal Activity of Dual Redox-Active Quinones: Trypanosoma cruzi Mitochondrion as a Target Organelle In Vitro and Anti-Inflammatory Properties In Vivo
Source: Pathogens. 2025 Dec 23;15(1):17. doi: 10.3390/pathogens15010017 (PMC12845226; doi:10.3390/pathogens15010017)
Supplement: Supplementary file 1 [file pathogens-15-00017-s001.zip › Duarte et al supplementary figures.pdf]

Supplementary figures

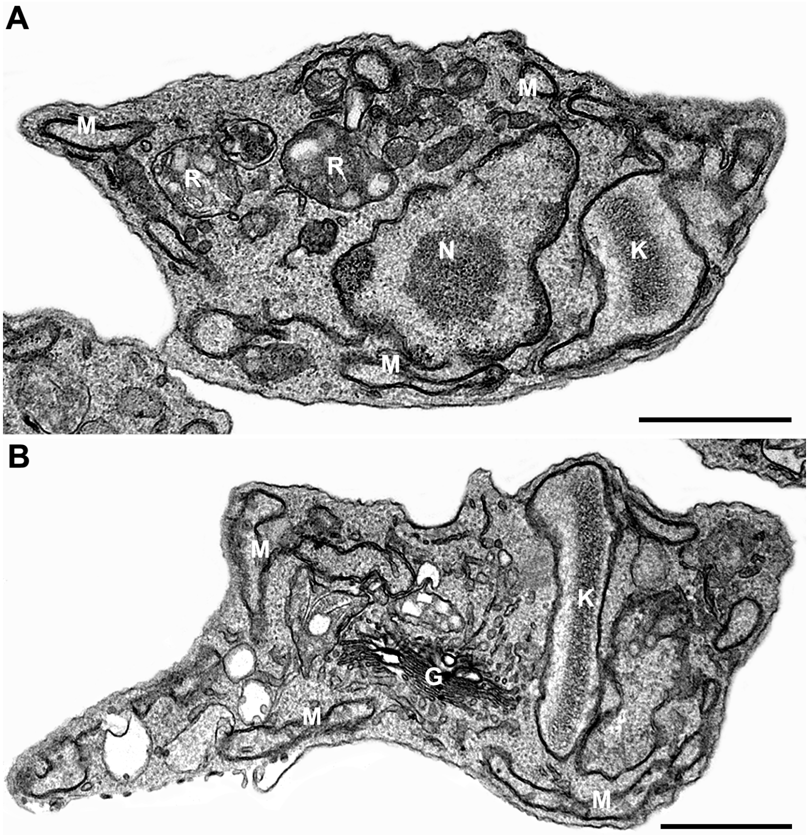

Figure S1. Ultrastructural analysis of *T. cruzi* epimastigotes treated with 0.4 μM NQ1 (**A**) and 0.9 μM NQ2 (**B**) *in vitro*. None of the naphthoquinones tested presented any morphological injury in the parasites. N: nucleus; M: mitochondria; R: reservosome; K: kinetoplast; G: Golgi. Bars = 0.5 μm.

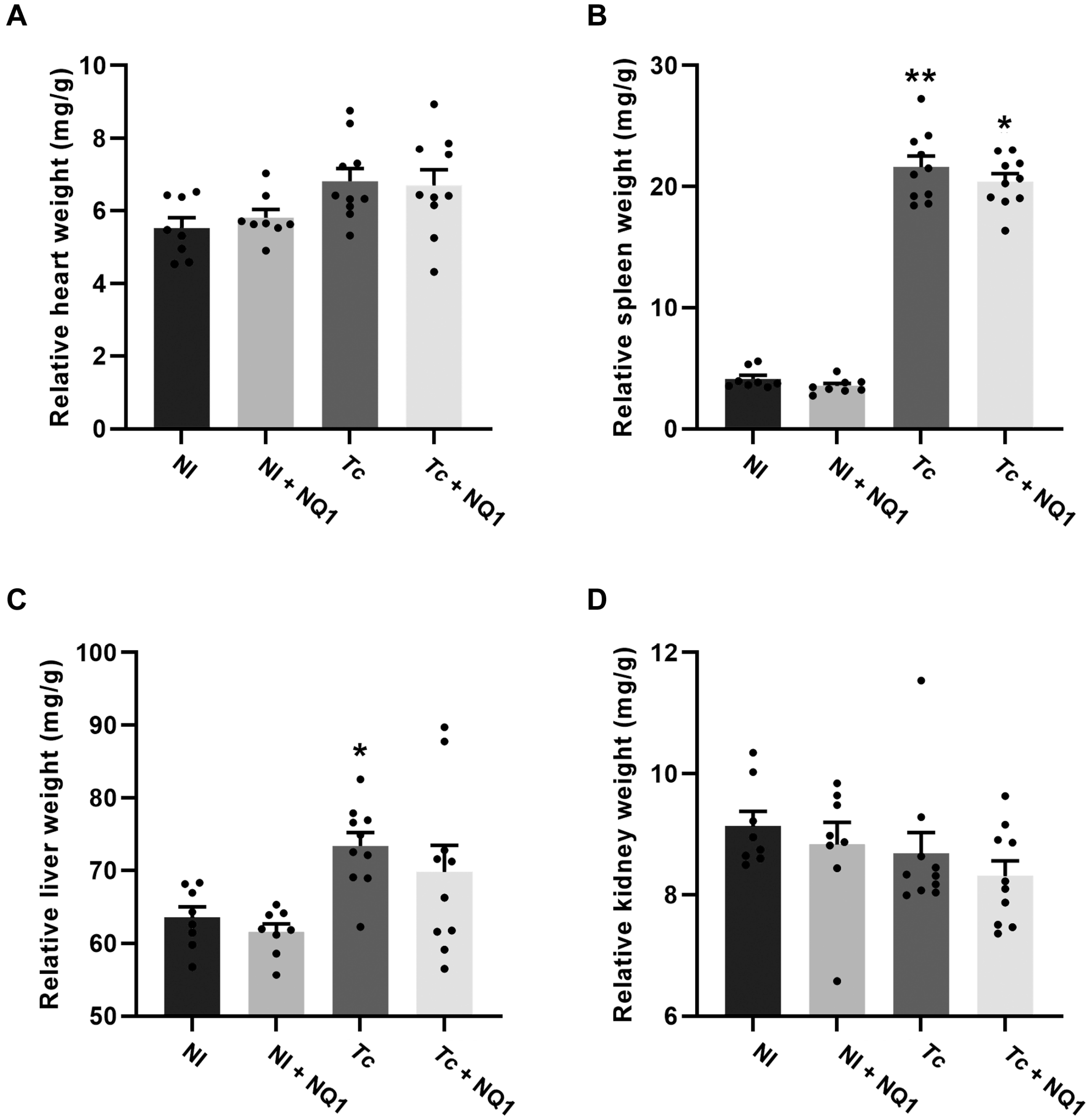

Figure S2. Effect of NQ1 (50 mg/kg) on the organs weight at 14 dpi. (**A**) Heart. (**B**) Spleen. (**C**) Liver. (**D**) Kidney. Relative weight: ratio organ and whole body weights. Mean ± standard deviation of at least 8 animals per group. Asterisks indicate significant differences between non-infected and infected groups (*P<0.05; ** P<0.01; *** P<0.001) by One-way ANOVA and Kruskal-Wallis tests. NI: non-infected; Tc: *T. cruzi*; dpi: days post infection.

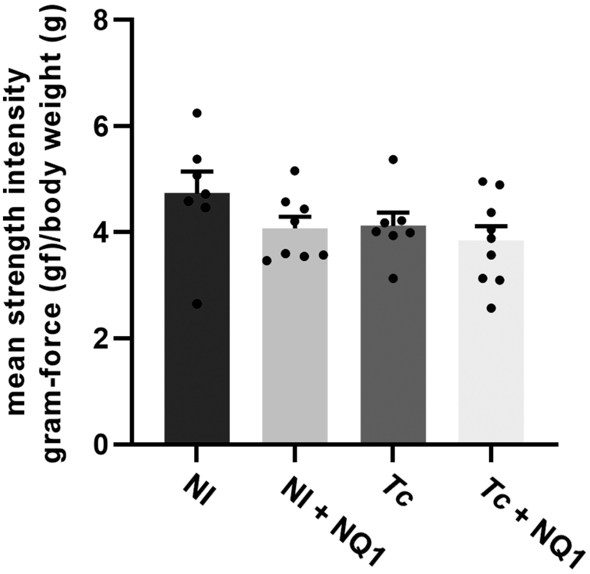

Figure S3. Effect of NQ1 (50 mg/kg) on the muscular strength at 14 dpi. Mean ± standard deviation of at least 8 animals per group. NI: non-infected; Tc: *T. cruzi*; dpi: days post infection.

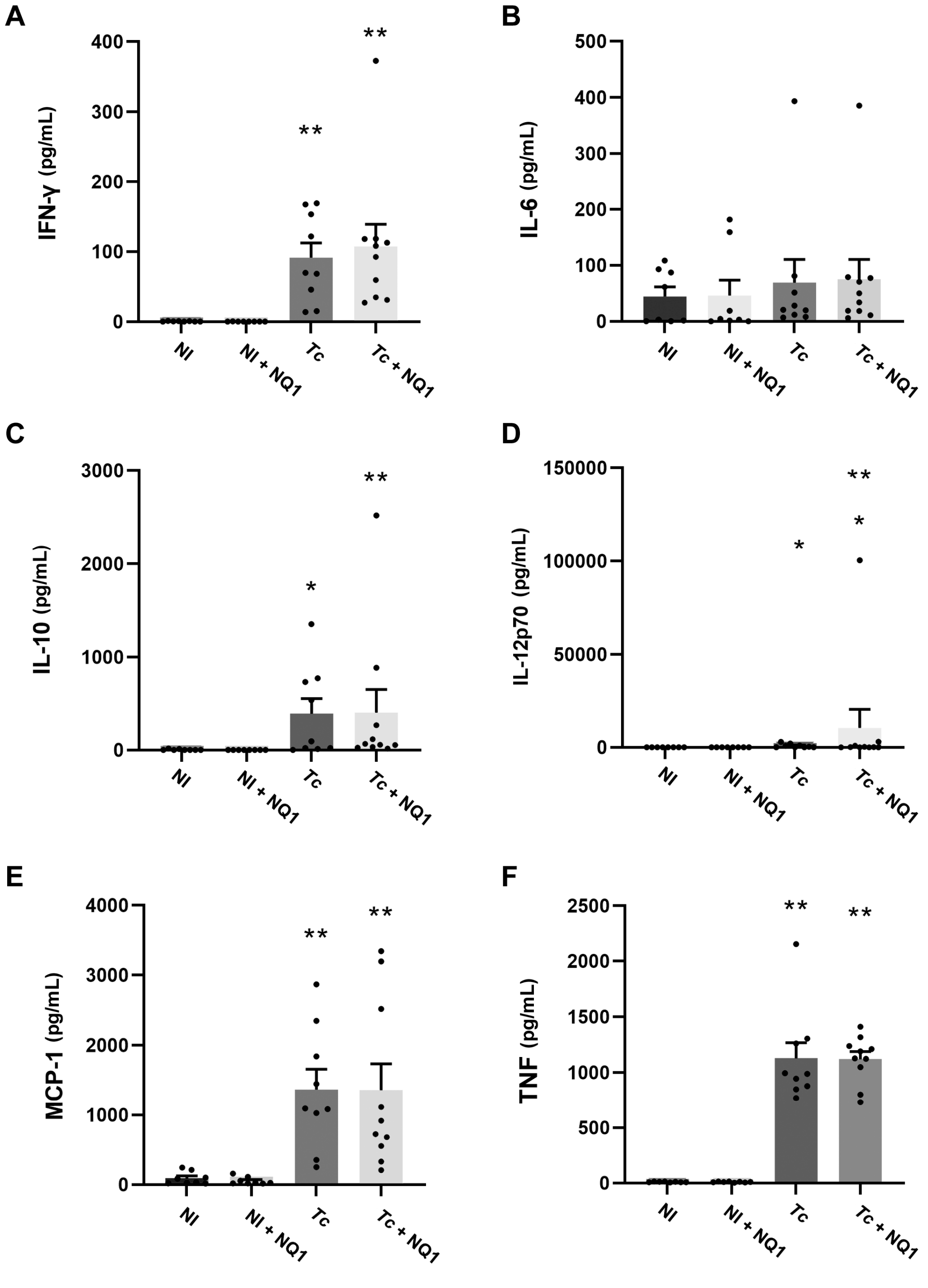

Figure S4. Effect of NQ1 (50 mg/kg) on the plasma levels of cytokines at 14 dpi. (**A**) IFN-ɣ; (**B**) IL-6; (**C**) IL-10; (**D**) IL-12p70; (**E**) MCP-1; (**F**) TNF. Mean ± standard deviation of at least 8 animals per group. Asterisks indicate significant differences between non-infected and infected groups (* P<0.05; ** P<0.01) by Kruskal-Wallis test. NI: non-infected; Tc: *T. cruzi*; dpi: days post infection.
