# Supplementary figures and images for: Trypanocidal Activity of Dual Redox-Active Quinones: Trypanosoma cruzi Mitochondrion as a Target Organelle In Vitro and Anti-Inflammatory Properties In Vivo
Source: Pathogens. 2025 Dec 23;15(1):17. doi: 10.3390/pathogens15010017 (PMC12845226; doi:10.3390/pathogens15010017)

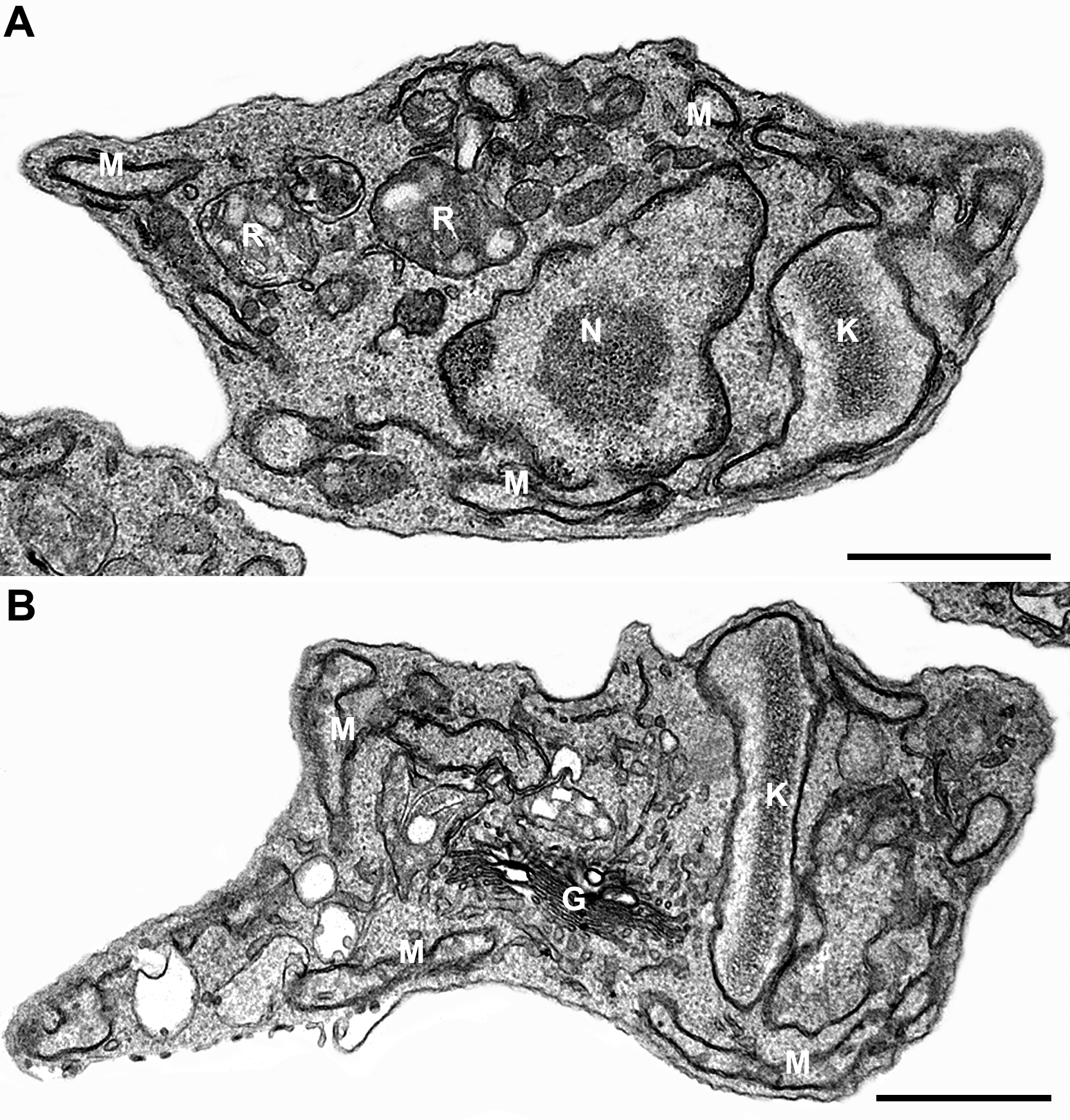

Supplement: Supplementary file 1 [file pathogens-15-00017-s001.zip › Duarte et al figure S1.tif]

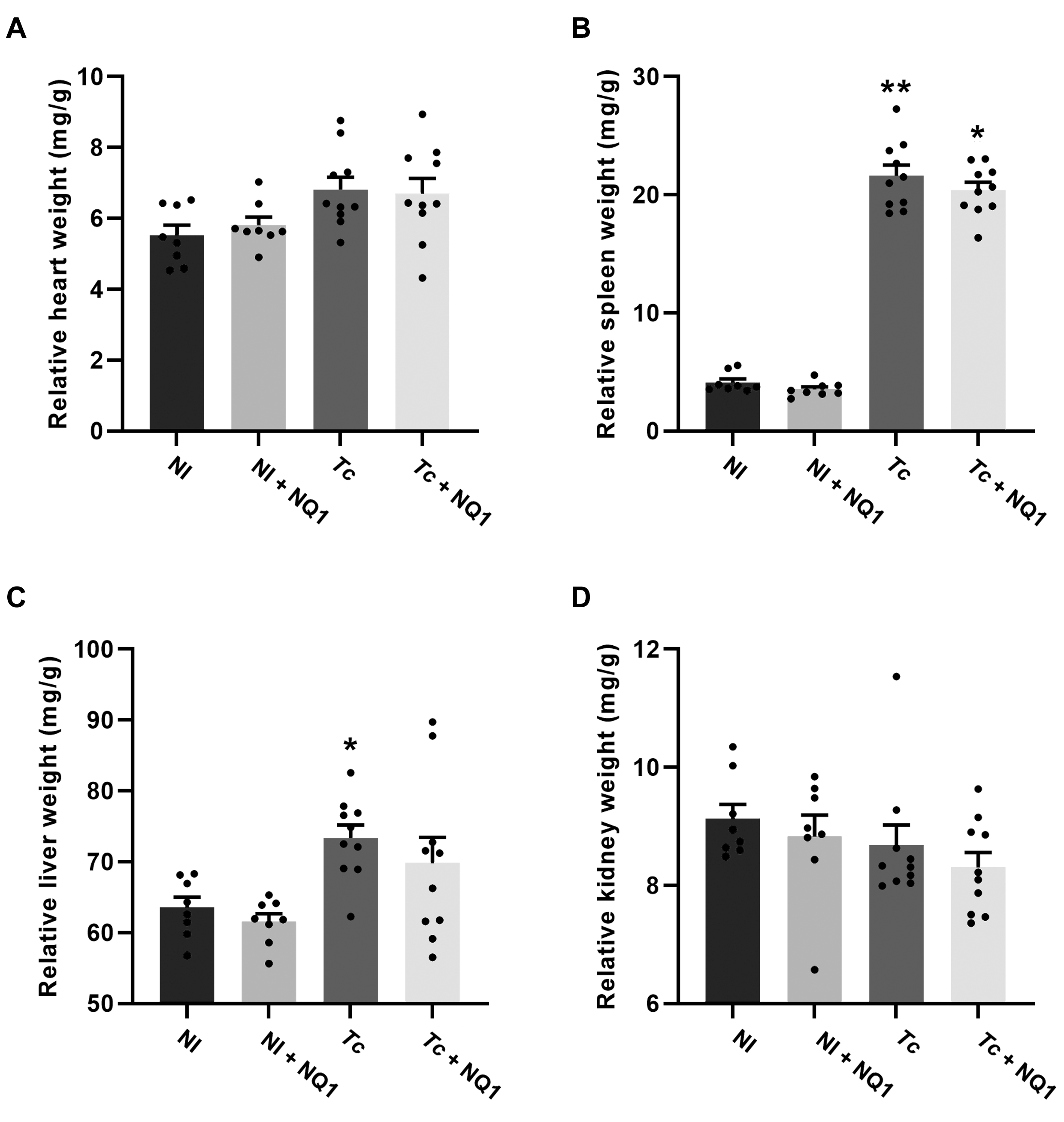

Supplement: Supplementary file 1 [file pathogens-15-00017-s001.zip › Duarte et al figure S2.tif]

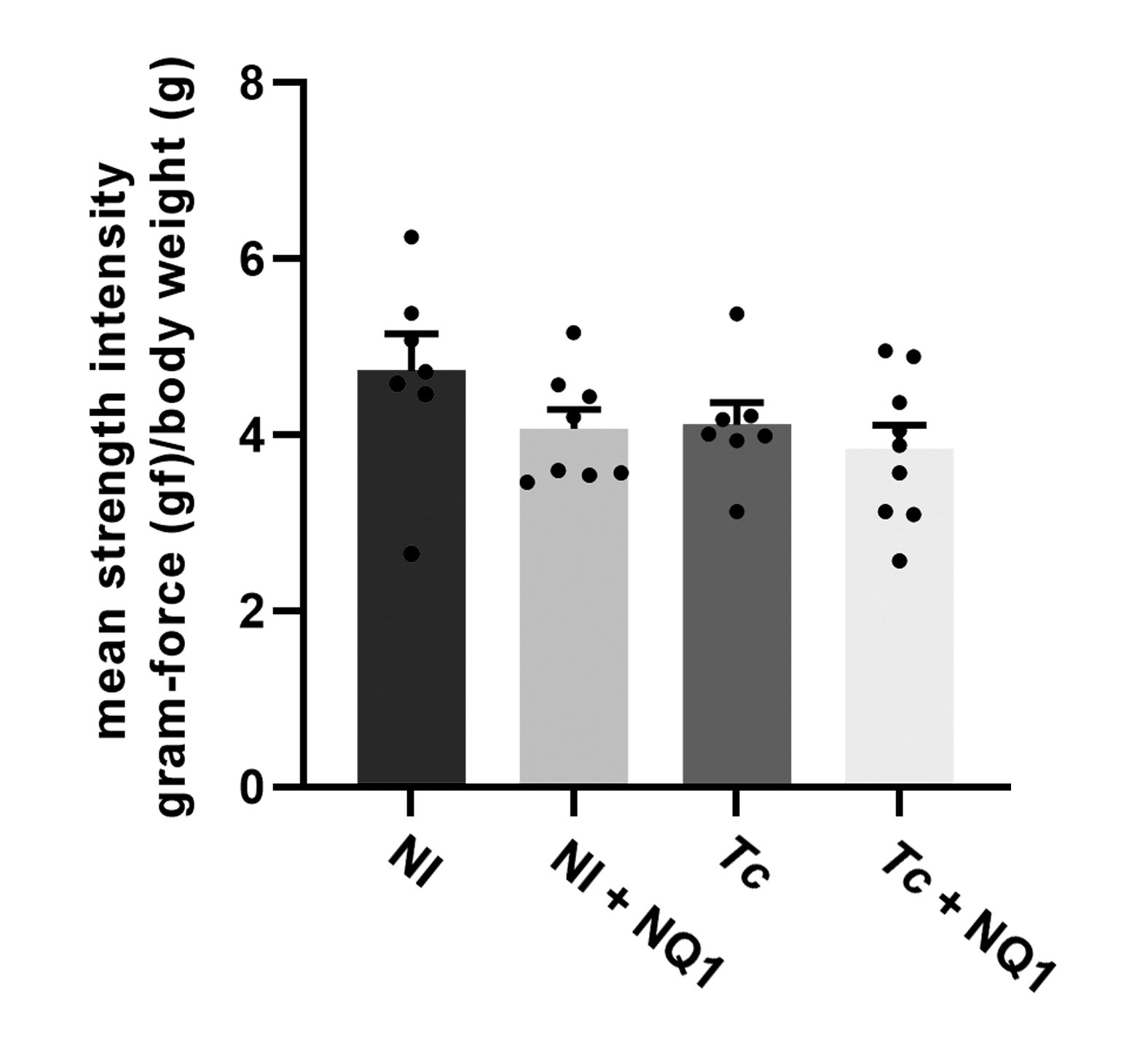

Supplement: Supplementary file 1 [file pathogens-15-00017-s001.zip › Duarte et al figure S3.tif]

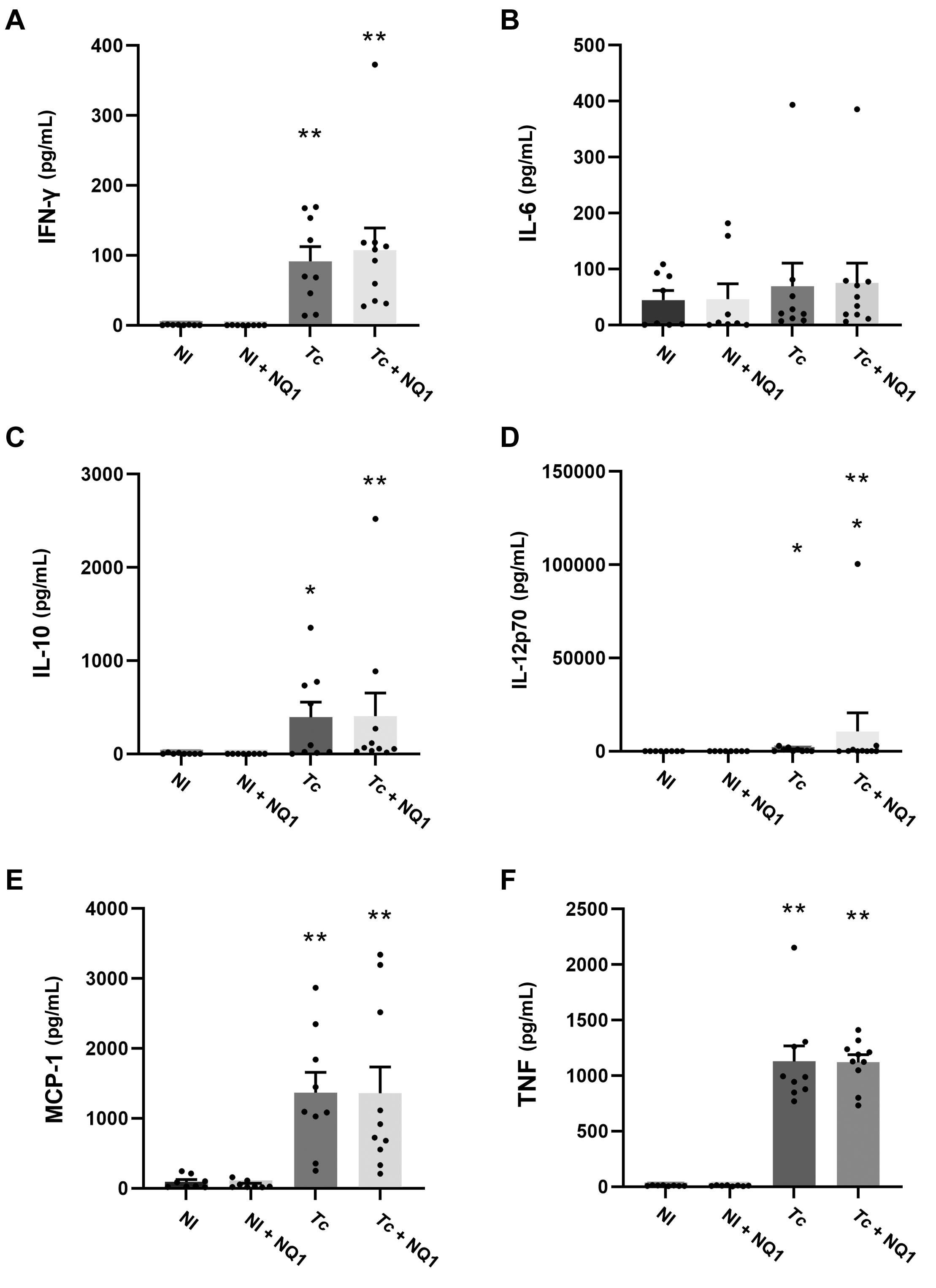

Supplement: Supplementary file 1 [file pathogens-15-00017-s001.zip › Duarte et al figure S4.tif]
